# New alleles of *nlp-2* , *nlp-22* , and *nlp-23* demonstrate that they are dispensable for stress-induced sleep in *C. elegans*

**DOI:** 10.17912/micropub.biology.001109

**Published:** 2024-02-01

**Authors:** Sage Aviles, Sanjita Subramanian, Matthew D Nelson

**Affiliations:** 1 Biology, Saint Joseph's University, Philadelphia, Pennsylvania, United States

## Abstract

Sleep is ancient and genetically conserved across phylogeny. Neuropeptide signaling plays a fundamental role in the regulation of sleep for mammals, fish, and invertebrates like *Caenorhabditis elegans* . Developmentally timed-sleep and stress-induced sleep of *C. elegans* are controlled by distinct and overlapping neuropeptide pathways. The RPamide neuropeptides *nlp-2* , *nlp-22* , and *nlp-23* , play antagonistic roles during the regulation of developmentally-timed sleep, however, their role in stress-induced sleep has not been explored. These genes are linked on the X chromosome, which has made genetic analyses challenging. Here we used CRISPR to generate new alleles of *nlp-22* and *nlp-23* , *nlp-22* ; *nlp-23* double mutants, and *nlp-2* ; *nlp-22* ; *nlp-23* triple mutants. Confirming previous studies, we find that *nlp-22* is required for developmentally-timed sleep, and show that *nlp-23* is also required. However, all three genes are dispensable for stress-induced sleep.

**Figure 1. The RPamide neuropeptides are dispensable for stress-induced sleep:**
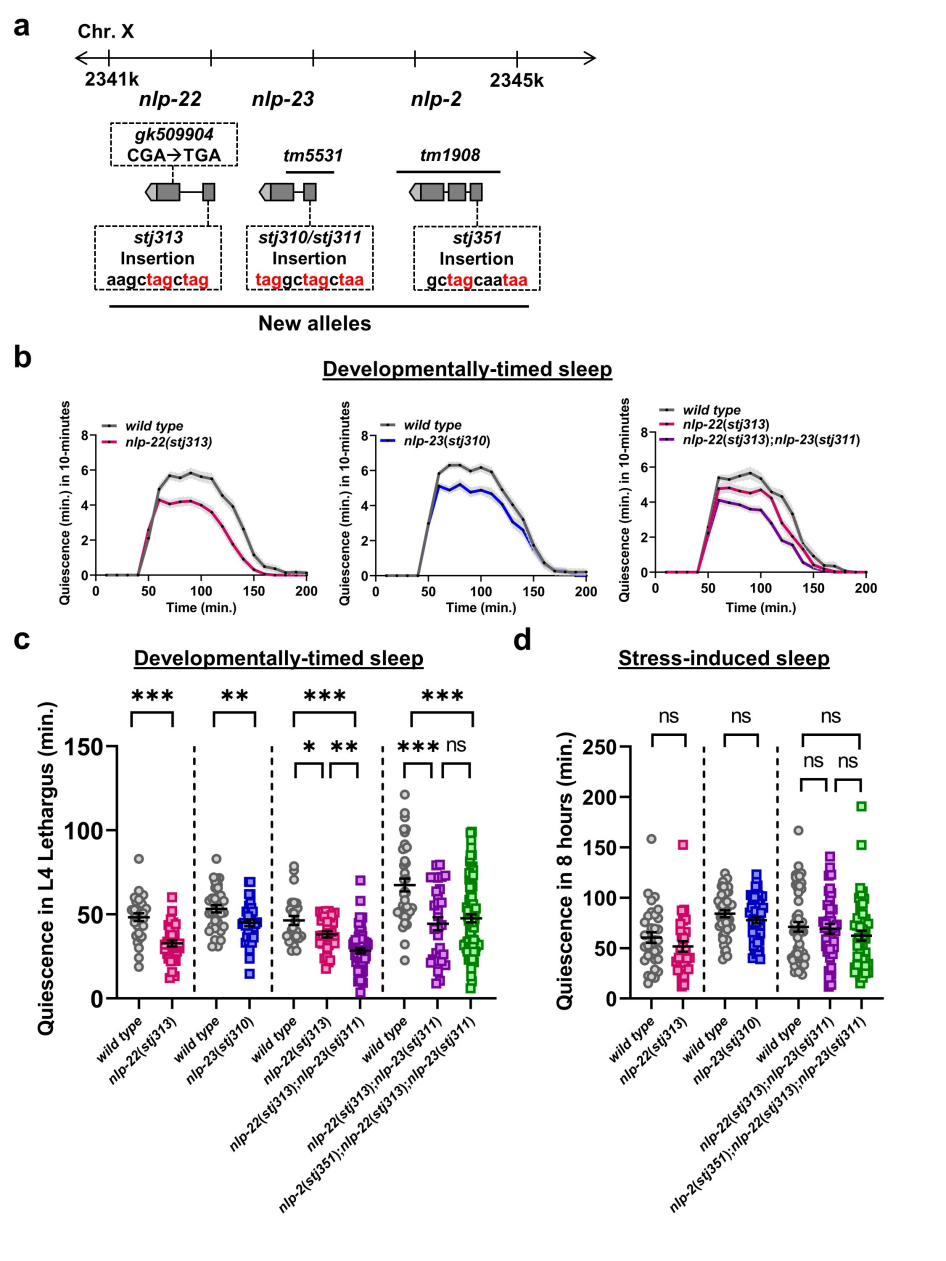
**(a)** Gene structures and alleles for the RPamide neuropeptides. **(b)** Average minutes of movement quiescence in 10-minute windows during L4 developmentally-timed sleep in wild-type (N=31) and *nlp-22* ( *stj313* ) (N=33) animals, wild-type (N=35) and *nlp-23* ( *stj310* ) (N=36) animals, and wild-type (N=26), *nlp-22* ( *stj313* ) (N=30), and *nlp-22* ( *stj313* ); *nlp-23* ( *stj311* ) (N=60) animals. **(c)** Total minutes of movement quiescence during L4 developmentally-timed sleep in wild-type (N=31) and *nlp-22* ( *stj313* ) (N=33) animals, wild-type (N=35) and *nlp-23* ( *stj310* ) (N=36) animals, wild-type (N=26), *nlp-22* ( *stj313* ) (N=30), and *nlp-22* ( *stj313* ); *nlp-23* ( *stj311* ) (N=60) animals, and wild-type (N=38), *nlp-22* ( *stj313* ); *nlp-23* ( *stj311* ) (N=34), and *nlp-2* ( *stj351* ); *nlp-22* ( *stj313* ); *nlp-23* ( *stj311* ) (N=82) animals. **(d)** Total minutes of movement quiescence during UV-induced sleep in wild-type (N=33) and *nlp-22* ( *stj313* ) (N=31) animals, wild-type (N=44) and *nlp-23* ( *stj310* ) (N=49) animals, wild-type (N=55), *nlp-22* ( *stj313* ); *nlp-23* ( *stj311* ) (N=41), and *nlp-2* ( *stj351* ); *nlp-22* ( *stj313* ); *nlp-23* ( *stj311* ) (N=50) animals. For both **(c)** and **(d)** statistical significance was calculated by Student’s t-test (2 genotypes) or one-way ANOVA followed by Tukey’s test (3 genotypes)(*p<0.05, **p<0.01, ***p<0.001).

## Description

Sleep is conserved across the animal kingdom, suggesting that its function is essential and the mechanisms evolutionarily ancient (Anafi et al. 2019) . The genetically-tractable roundworm *Caenorhabditis elegans* displays multiple forms of sleep, with the two most well-studied being developmentally-timed sleep (Raizen et al. 2008) and stress-induced sleep (Hill et al. 2014) . Developmentally-timed sleep takes place during larval transitions, a life-stage termed lethargus (Singh and Sulston 1978) , which is immediately followed by ecdysis (i.e., molting of the exoskeleton) (Singh and Sulston 1978, Trojanowski et al. 2015) . Behaviors, physiological characteristics, and the molecular regulation suggest that developmentally-timed sleep is related to the circadian-sleep of insects and mammals (Trojanowski and Raizen 2016) , and thus fulfills the widely-accepted definitions of sleep (Campbell and Tobler 1984, Raizen et al. 2008) . These include periods of reversible quiescence, decreased sensory arousal (Raizen et al. 2008) , a stereotypic posture (Schwarz et al. 2012, Tramm et al. 2014) , homeostatic sleep drive following deprivation (Raizen et al. 2008, Nagy et al. 2014) , lethality in response to chronic deprivation (Driver et al. 2013) , and regulation by a molecular clock (Jeon et al. 1999, Monsalve et al. 2011) . Like in more complex animals (Crocker and Sehgal 2010) , neuropeptide signaling plays a central role in the regulation of developmentally-timed sleep. Specifically, sleep behavior requires the neuropeptides *nlp-22* (Nelson et al. 2013) and *flp-11* (Turek et al. 2016) , whereas arousal is mediated by *nlp-2* (Van der Auwera et al. 2020) , *pdf-1* (Choi et al. 2013) , and *flp-2* (Chen et al. 2016) . While the cognate receptors and downstream circuitry for some of these peptides have been identified, the mechanisms that regulate sleep behavior are still being determined.

In contrast, stress-induced sleep occurs at any life stage in response to noxious stimuli which damage cells such as extreme temperature, wounding, infection, ultraviolet (UV) irradiation, hyperosmotic conditions, and ethanol toxicity (Hill et al. 2014, DeBardeleben et al. 2017, Goetting et al. 2020, Sinner et al. 2021) . Stress-induced sleep also fulfills the behavioral definitions of sleep; however, it lacks a circadian component (Campbell and Tobler 1984, Hill et al. 2014) . Stress-induced sleep is regulated by a collection of neuropeptides. First, sleep behavior requires epidermal growth factor (EGF) peptides, which are encoded by *lin-3* , and the EGF receptor *let-23* which is required specifically in the neuropeptidergic interneurons ALA (Van Buskirk and Sternberg 2007, Hill et al. 2014) and RIS (Konietzka et al. 2020) . The ALA expresses numerous neuropeptide genes, such as *flp-13* , *flp-24* , *nlp-8* , *nlp-14* , and others, that are required for quiescence of movement, feeding, and defecation (Nelson et al. 2014, Nath et al. 2016, Honer et al. 2020) . The RIS expresses *flp-11* , required for movement quiescence (Konietzka et al. 2020) . Like with developmentally-timed sleep, how these various peptides precisely modulate behavior is unclear.

Some of these genes, such as *flp-11* and *nlp-14* , are required for both sleep states (Turek et al. 2013, Honer et al. 2020, Konietzka et al. 2020) , as is the neuropeptide receptor *npr-38* (Le et al. 2023) . However, it is unclear if other neuropeptide pathways are required for both forms of sleep. Here, we tested this for the RPamide neuropeptides encoded by *nlp-2* , *nlp-22* , and *nlp-23* . RPamides share a C-terminal amino acid motif of arginine (R), and proline (P). In most of these peptides, the RP sequence is followed by a glycine (G), which serves as a target for amidation, thus the name RPamides (Nathoo et al. 2001, Van der Auwera et al. 2020) . Although not the focus of this study, it should be noted that *nlp-46* also encodes a peptide with a c-terminal RPG motif, therefore it may represent another member of the RPamides (McVeigh et al. 2008, Van Bael et al. 2018, Van der Auwera et al. 2020) . The *nlp-2* , *nlp-22* , and *nlp-23* genes are located within a 3500 base pair region on the X chromosome. In previous work, movement quiescence during developmentally-timed sleep was reduced in *nlp-22* ( *gk509904)* mutant animals and in animals treated with *nlp-22* RNAi (Nelson et al. 2013) . The *gk509904* allele is a point mutation that introduces a stop codon prior to the encoded peptide (Thompson et al. 2013) , thus likely represents a null. A reduction in developmentally-timed sleep was not detected in *nlp-23* ( *tm5531* ) deletion mutants, however, the sample size was low in this study (N=6) (Van der Auwera et al. 2020) . The *tm5531* allele is a deletion that removes the signal peptide, thus is also likely a null. In contrast, *nlp-2* ( *tm1908* ) deletion mutants, in which the entire *nlp-2* gene is deleted, displayed increased levels of movement quiescence during developmentally-timed sleep (Van der Auwera et al. 2020) , suggesting that *nlp-2* peptides are required for arousal. In each of these instances, single mutants were analyzed. To better understand the roles of the RPamides during sleep, we used a CRISPR approach (Paix et al. 2017) to generate new loss-of-function alleles of *nlp-22* and *nlp-23* , and *nlp-22* ; *nlp-23* double, and *nlp-2* ; *nlp-22* ; *nlp-23* triple mutants ( **Figure 1a** ).

First, we measured movement quiescence during developmentally-timed sleep, using the WorMotel (Churgin et al. 2017) , in *nlp-22* ( *stj313* ) and *nlp-23* ( *stj310* ) animals and found that quiescence was reduced in both backgrounds ( **Figure 1b and 1c** ). This validates previous work with *nlp-22* (Nelson et al. 2013) , however, contradicts prior work with *nlp-23* (Van der Auwera et al. 2020) . One explanation of this discrepancy is that the small sample size of the initial study (Van der Auwera et al. 2020) did not allow for the detection of this relatively subtle difference in movement quiescence. Additionally, the two *nlp-23* strains were generated using different methodologies (i.e., random mutagenesis vs. CRISPR), thus background mutations in the *tm5531* strain may suppress the effects of removing *nlp-23* . Last, the methods employed when measuring quiescence were different between the two studies; this may also contribute to the discrepancy of phenotypes. Next, developmentally-timed sleep was compared between wild-type, *nlp-22* ( *stj313* ), and *nlp-22* ( *stj313* ); *nlp-23* ( *stj311* ) animals. Quiescence was significantly lower in the double mutants compared to the *nlp-22* single mutants, suggesting that *nlp-22* and *nlp-23* work in an additive manner during developmentally-timed sleep ( **Figure 1b and 1c** ). Last, we examined wild-type, *nlp-22* ( *stj313* ); *nlp-23* ( *stj311* ), and *nlp-2* ( *stj351* ) *; nlp-22* ( *stj313* ); *nlp-23* ( *stj311* ) animals, however, movement quiescence was not significantly different between the double and triple mutants ( **Figure 1c** ). Considering *nlp-2* ( *tm1908* ) deletion mutants displayed increased quiescence (Van der Auwera et al. 2020) , our data would suggest that *nlp-22* and *nlp-23* function downstream of *nlp-2* . However, this was not specifically tested in this study. Taken together, our data suggest that *nlp-22* and *nlp-23* are required for developmentally-timed sleep, and suggest that these phenotypes over-ride the effects of removing *nlp-2* alone.

To test the requirement for the RPamides during stress-induced sleep, animals were exposed to ultraviolet irradiation (i.e., UV-induced sleep), as described (DeBardeleben et al. 2017) , and movement quiescence was measured using the WorMotel (Churgin et al. 2017) . UV-induced sleep was compared between *nlp-22* ( *stj313* ) and wild-type animals, however, no significant difference was observed ( **Figure 1d** ). Also, no difference was detected between wild-type and *nlp-23* ( *stj310* ) animals ( **Figure 1d** ). Next, we compared UV-induced quiescence between wild-type, *nlp-22* ( *stj313* ); *nlp-23* ( *stj311* ), and *nlp-2* ( *stj351* ) *; nlp-22* ( *stj313* ); *nlp-23* ( *stj311* ) animals. Once again, no differences were observed between any of these genotypes ( **Figure 1d** ). These data demonstrate that the RPamides are dispensable for stress-induced sleep in response to UV exposure. More broadly, these data suggest that the roles of the RPamide neuropeptides *nlp-2* , *nlp-22* , and *nlp-23* are specific to developmentally-timed sleep and further demonstrate (Trojanowski et al. 2015) , that a subset of neuropeptide pathways regulate both forms of sleep, while others play narrower roles.

## Methods

**Worm maintenance and strains**

*C. elegans* strains used in this study are listed in the reagents table. All animals were maintained at 20°Celsius on agar plates containing nematode growth medium and fed the OP50 derivative bacterial strain DA837 (Davis et al. 1995) .

**Construction of mutants**

SJU310, SJU313, SJU346, and SJU373 were constructed by CRISPR/Cas9 gene editing, using a published protocol (Arribere et al. 2014) . To produce loss-of-function alleles, insertions were generated that contained multiple stop codons and an NheI restriction enzyme site, 3’ of the encoded signal peptide. An edit of the *dpy-10* gene was made which resulted in an easily identifiable dumpy (dpy) or roller (rol) phenotype, to allow for screening. A mixture of guide RNA (gRNA) duplexed with Alt-R ® CRISPR-Cas9 tracrRNA (IDT ©), Alt-R ® S.p. Cas9 Nuclease V3 (IDT ©) and, oligonucleotide repair templates were injected into day-1 adult wild-type animals to generate mutant strains SJU310 *nlp-23* ( *stj310* ) and SJU313 *nlp-22* ( *stj313* ) mutants. To generate the double mutant strain SJU346 *nlp-22* ( *stj313* ); *nlp-23* ( *stj311* ), reagents to make the *stj310* allele were injected into SJU313 animals. Although *stj310* and *stj311* are identical insertions for *nlp-23* , they were given different names because they were made by independent injections. To construct the triple mutant strain SJU373 *nlp-2* ( *stj351* ); *nlp-22* ( *stj313* ); *nlp-23* ( *stj311* ), *nlp-2* gRNA and repair templates were injected into SJU346 animals. In each case, dpy or rol progeny of the injected animals were transferred to individual plates and maintained to the next generation. Worm lysates were made from each plate and used as templates for PCR to amplify a portion of the edited gene. The amplicon was treated with NheI restriction enzyme and analyzed by agarose gel electrophoresis. Once a strain was isolated and the *dpy-10* mutations were removed by random segregation (rol phenotypes) or crossing with N2 (dpy phenotypes), alleles were confirmed by sequencing (Genewiz ©). Custom gRNA, repair templates, and screening primers are listed in the reagents table.

**WorMotel behavioral assays**

Movement quiescence was quantified using the WorMotel, as previously described (Churgin et al. 2017) . For developmentally-timed sleep, L4 animals that were actively feeding were transferred to the agar surfaces of 24-welled polydimethylsiloxane (PDMS) microchips. Images were captured every 10 seconds for 12 hours. Lethargus was identified as a period of time in which the movement quiescence was above 0.5 minutes in a 10-minute window, and was sustained for at least 20 minutes ( **Figure 1b** ). Total quiescence was determined and averaged over multiple trials for each genotype. For stress-induced sleep, first-day adults were picked onto the agar surfaces of 24-welled PDMS microchips. The chip was placed into a UV-cross linker (Ultraviolet, 254 UVP) and exposed to 1500 J/m ^2^ of UV light. Images were captured every 10 seconds for 8 hours and total minutes of quiescence was determined. For both forms of sleep, when two genotypes were analyzed in the same experiment, the averages were compared by Student’s t-test. If three genotypes were imaged simultaneously then the averages were compared by one-way ANOVA followed by Tukey’s multiple comparisons test.

## Reagents

**Table:** 

**Strain**	**Genotype**	**Available from**
N2	Bristol (Wild type)	CGC
SJU310	*nlp-23* ( *stj310* )	Nelson Lab
SJU313	*nlp-22* ( *stj313* )	Nelson Lab
SJU346	*nlp-22* ( *stj313* ); *nlp-23* ( *stj311* )	Nelson Lab
SJU373	*nlp-2* ( *stj351* ); *nlp-22* ( *stj313* ); *nlp-23* ( *stj311* )	Nelson Lab
**Reagent**	**Sequence**	**Description**
oSJUcrRNA24	CGTTCCATAATCGTCTTCATCGG	gRNA for *nlp-22* ( *stj313* )
oSJUcrDNA57	CTTTCCCAACTCGGAAATGCGTTCCATAATCGTCTaagctagctagTCATCGGATTGACGATCTTCGCGTTGGACATTCTT	Repair template for *nlp-22* ( *stj313* )
oSJUcrDNA66	GTTCACAAAACCGAGAGCAAC	Forward screening primer for *nlp-22*
oSJUcrDNA67	GAAGACATCGATTCCACCCTG	Reverse screening primer for *nlp-22*
oSJUcrRNA24	CCTCGTCATTTGGATGGCACTTC	gRNA for *nlp-23* ( *stj310* )
oSJUcrDNA59	TATCACTTTCAAAGTCAATGGCAGCTCACCTCGTCtaggctagctaaATTTGGATGGCACTTCTTGGAGTCTCAGCTCATGC	Repair template for *nlp-23* ( *stj310* ) and *stj311*
oSJUcrDNA62	GATACACCTATAGTCGTTGTATTC	Forward screening primer for *nlp-23*
oSJUcrDNA63	CTCTCTGCAAATGGCATTGATC	Reverse screening primer for *nlp-23*
oSJUcrRNA25	CCGCTTCAGGTCTATCGTCCTGA	gRNA for *nlp-2* ( *stj351* )
oSJUcrDNA60	GCTCTGCGCAGTTTATTCTGAAGCAGTTCCGCTTCgctagcaataaAGGTCTATCGTCCTGACGAATCATCGGTTAGTGGA	Repair template for *nlp-2* ( *stj351* )
oSJUcrDNA64	CTCGTTATCAATATTCCCACTG	Forward screening primer for *nlp-2*
oSJUcrDNA65	CATTGATCGTTTCATGATGAG	Reverse screening primer for *nlp-2*
